# Extraction of Gallic Acid and Ferulic Acid for Application in Hair Supplements

**DOI:** 10.3390/molecules28052369

**Published:** 2023-03-04

**Authors:** Pedro Velho, Catarina S. Rebelo, Eugénia A. Macedo

**Affiliations:** 1 LSRE-LCM—Laboratory of Separation and Reaction Engineering—Laboratory of Catalysis and Materials, Faculty of Engineering, University of Porto, Rua Dr. Roberto Frias, 4200-465 Porto, Portugal; 2 ALiCE—Associate Laboratory in Chemical Engineering, Faculty of Engineering, University of Porto, Rua Dr. Roberto Frias, 4200-465 Porto, Portugal

**Keywords:** hair loss, gallic acid, ferulic acid, ethyl lactate, ATPS

## Abstract

Food supplements based on antioxidants and vitamins are often prescribed to correct inefficiencies in the human diet and delay diseases such as premature aging and alopecia (temporary or permanent hair loss), given the free radical scavenging activity of these biomolecules. By reducing the concentration of reactive oxygen species (ROS), which promote abnormal hair follicle cycling and morphology, follicle inflammation and oxidative stress are reduced, minimising the effects of these health issues. Gallic acid (GA), which is significantly present in gallnuts and in pomegranate root bark, and ferulic acid (FA), commonly found in brown rice and coffee seeds, are very important antioxidants for the preservation of hair colour, strength and growth. In this work, these two secondary phenolic metabolites were successfully extracted in the Aqueous Two-Phase Systems (ATPS) {ethyl lactate (1) + trisodium citrate (2) + water (3)} and {ethyl lactate (1) + tripotassium citrate (2) + water (3)} at 298.15 K and 0.1 MPa, moving towards the application of these ternary systems in extracting antioxidants from biowaste and their *a posteriori* processing as food supplements for hair fortification. The studied ATPS provided biocompatible and sustainable media for the extraction of gallic acid and ferulic acid, yielding low mass losses (<3%) and contributing to an eco-friendlier production of therapeutics. The most promising results were obtained for ferulic acid, which attained maximum partition coefficients (*K*) of 15 ± 5 and (3 ± 2) · 10^1^ and maximum extraction efficiencies (*E*) of (92.7 ± 0.4)% and (96.7 ± 0.4)% for the longest tie-lines (TLL = 69.68 and 77.66 *m*%) in {ethyl lactate (1) + trisodium citrate (2) + water (3)} and {ethyl lactate (1) + tripotassium citrate (2) + water (3)}, respectively. Moreover, the effect of pH on the UV-Vis absorbance spectra was studied for all the biomolecules to minimise errors in solute quantification. Both GA and FA were found to be stable at the used extractive conditions.

## 1. Introduction

Alopecia is a common health issue characterised by temporary or permanent hair loss. It is caused by abnormal hair follicle cycling and morphology, and frequently has a dramatic influence on human self-esteem and quality of life, even though it is not a life-threatening disease [1,2,3]. The treatment of alopecia is sometimes impossible due to genetic predisposition, advanced age and the current lack of knowledge concerning the molecular pathways that control normal hair follicle growth and cycling [4,5]. However, food supplementation by minerals, vitamins and antioxidants is often applied to slow down hair loss by correcting dietary inefficiencies which lead to the production of reactive oxygen species (ROS) [6]. ROS promote a state of inflammation and oxidative stress which harms hair growth, so the consumption of natural chemical compounds with free radical scavenging activity, such as polyphenols, phytosterols, phytoestrogens, fatty acids and terpenoids, helps to prevent alopecia by blocking the harmful effects of ROS and of some hormones such as androgen [4,7].

Antioxidants are chemical species that, when present at low concentrations, significantly delay or prevent the oxidation of bioorganic materials by neutralising the reactive oxygen species with the donation or acceptance of electrons, which revert the unpaired status of the free radicals, thereafter reducing their activity [8]. Polyphenols, i.e., a chemical family of powerful antioxidants, compose the most abundant bioactive nutrient chemicals in plants (phytochemicals) and are thought to play a significant role in the prevention of neurodegenerative and cardiovascular diseases [9,10,11]. Moreover, polyphenols exhibit strong anti-inflammatory, anti-osteoporotic, anti-cancer and anti-viral properties [9,11,12,13].

Gallic acid (GA) is a phenolic compound present in black and green tea, gallnuts, pomegranates, oranges, grapes and berries, and is commonly used as a food additive [14,15]. Gallic acid is a benzoic acid of great importance due to its antiulcerogenic, antimicrobial, antifungal and antioxidant properties [14,16] and has been successfully extracted using, for example, ionic liquids [17,18] and ethanol [19,20]. Moreover, GA is a building block for some important active pharmaceutical ingredients (APIs), such as trimethoprim (antibiotic), podophyllotoxin (for skin warts treatment), and colchicine (for gout treatment) [14,21].

Ferulic acid (FA) is one of the most abundant phenolic compounds in plants, and can be found covalently conjugated to the cell walls in tomatoes, sweet corn, brown rice, grapes, olives and coffee seeds [22,23]. Besides its antioxidant, anticancer, antimicrobial, antiallergic and anti-inflammatory activities, it is also a precursor in the synthesis of useful aromatic chemical compounds, such as vanillin (a flavouring agent used in foods and beverages) [24,25]. So far, different techniques have been applied to obtain FA, including enzymatic, alkaline, and acidic extractions, which often fail to preserve the bioactivity of this antioxidant [22].

GA and FA are very important to the preservation of hair colour, strength and growth, but their extraction from natural matrices is hindered by substrate diversity and solute sensitivity towards oxidation and hydrolysis [22], so there is a high demand in the pharmaceutical and cosmetic industries for compatible extractive media. Aqueous Two-Phase Systems (ATPS), or Aqueous Biphasic Systems (ABS), constitute a benign liquid-liquid fractionation technique aimed at the efficient extraction of, for example, proteins [26,27,28], antioxidants [11,29,30,31], vitamins [8,30,32], pigments [33,34] and drugs [35,36], which has been gaining increased attention over the years [37]. These systems are formed by mixing water-soluble components, such as polymers and salts, in a water-rich medium at concentrations which create two immiscible phases: the top and the bottom phases [31]. When equilibrium is attained, each phase becomes significantly richer than the other in one of the water-soluble components, causing considerable asymmetries between the top and bottom phases in properties such as polarity, density, hydrophobicity and viscosity [31], which will rule the migration (partition) of eventual solutes between the phases. ATPS are considered simple to scale-up, provide non-toxic and biocompatible extractive media and allow high recovery yields to be obtained, so they became popular replacements for classic organic solvents, which are more volatile, toxic, and less secure [17,38].

Nowadays, eco-friendlier solvents compatible with application in ATPS have emerged. One of the most widely studied green solvents in the extraction of biomolecules is ethyl lactate (EL), which is a bio-renewable and biodegradable solvent with low toxicity towards humans and animals [39]. Ethyl lactate is produced by the esterification reaction between ethanol and lactic acid, which can be produced from biomass fermentation [40,41]. EL is known to form ATPS with some organic salts (for example, citrates and tartrates [11,29]), which are generally preferred to inorganic salts due to the higher biocompatibility of the former. Generally, ATPS with citrate-based salts, such as sodium citrate (Na_3_Citrate) and potassium citrate (K_3_Citrate), tend to obtain higher recovery yields in the extraction of biomolecules [42] and are known for their application as additives in food products, contributing to pH control, acidity regulation, flavour enhancement and preservation [42,43,44].

In this work, partition studies were carried out for gallic acid (GA) and ferulic acid (FA) in two biodegradable Aqueous Two-Phase Systems (ATPS), {ethyl lactate (1) + trisodium citrate (2) + water (3)} and {ethyl lactate (1) + tripotassium citrate (2) + water (3)}, at 298.15 K and 0.1 MPa. The final goal was to find suitable ternary systems to extract antioxidants from biowaste and contribute towards their *a posteriori* implementation as food supplements for hair fortification.

## 2. Materials and Methods

### 2.1. Chemicals

The list of chemicals and their respective supplying companies, purities, Chemical Abstracts Service (CAS) numbers and abbreviations can be seen in Table 1. No additional purification steps nor pre-treatments were carried out.

### 2.2. Apparatus and Experimental Procedure

#### 2.2.1. Effect of pH in the UV-Vis Absorbance Spectra

To study the effect of pH on the ultraviolet-visible (UV-Vis) absorbance spectra of gallic acid (GA) and ferulic acid (FA), the mean electrical charge (q) of each biomolecule was calculated as a function of pH, using the negative base-10 logarithm of their acid dissociation constants $\left(pK_{a}\right)$: 4.28, 8.62 and 11.90 for GA [45], and 4.50 and 8.92 [46] for FA. Since these particular phenolic compounds may present acidic behaviour at favourable pH values, the relative abundance of a certain acidic stage with respect to its conjugate base was calculated following [8,47]:

(1) $$\frac{\left[A^{q_{0}-i+1}\right]}{\left[A^{q_{0}-i}\right]}=10^{{\mathrm{pH}}_{\mathrm{phase}}-pK_{a}^{i}}$$

where $q_{0}$ is the initial electrical charge (at pH=0), i is the number of the dissociation constant ($pK_{a}^{i}$) under observation, ${\mathrm{pH}}_{\mathrm{phase}}$ is the pH of the phase, and $\left[A^{q_{0}-i+1}\right]$ and $\left[A^{q_{0}-i}\right]$ are the mole concentrations of the antioxidant species with electrical charges equal to $\left(q_{0}-i+1\right)$ e and ($q_{0}-i$) e, respectively, and e stands for the elementary charge (1.602∙10^−19^ C).

Once the relative abundances were calculated for each conjugate acid-base pair, the mole fraction of each antioxidant species with an electrical charge equal to $\left(q_{0}-i+1\right)$ e was calculated using [47]:

(2) $$x_{A^{q_{0}-i+1}}=\frac{\left[A^{q_{0}-i+1}\right]}{\left[A^{q_{0}-1}\right]}/\left(\frac{\left[A^{q_{0}}\right]}{\left[A^{q_{0}-1}\right]}+1+\overset{i_{\max}}{\underset{j=2}{\sum }}\left[\overset{j}{\underset{k=2}{\prod }}\frac{\left[A^{q_{0}-k}\right]}{\left[A^{q_{0}-\left(k-1\right)}\right]}\right]\right)$$

where i is the number of the dissociation constant under observation and $i_{\max}$ is the maximum number of protons (H^+^) the antioxidant can donate.

Then, the mean electrical charge of the antioxidant in solution (q) was determined by the weighted arithmetic mean given by Equation (3) [47].

(3) $$q=\overset{i_{\max}}{\underset{i=1}{\sum }}\left[x_{A^{q_{0}-i+1}}\cdot \left(q_{0}-i+1\right)\right]+\left[1-\overset{i_{\max}}{\underset{i=1}{\sum }}\left(x_{A^{q_{0}-i+1}}\right)\right]\cdot \left(q_{0}-i_{\max}\right)$$

Afterwards, five aqueous solutions of gallic acid $\left(\sim 1.5\cdot 10^{-4}\;g\cdot {\mathrm{mL}}^{-1}\right)$ and ferulic acid $\left(\sim 8.0\cdot 10^{-5}\;g\cdot {\mathrm{mL}}^{-1}\right)$ were prepared at pH conditions near integer values of q (to allow determining the UV-Vis absorbance spectra of each differently-charged antioxidant species) or near the known pH values of the applied ATPS (to analyse a distribution of antioxidant species which is similar to the future extractive conditions). To do so, mass (m) determinations were performed with an ADAM AAA 250L balance with a measurement uncertainty of ±10^−4^ g, and pH was evaluated with a Crison pH meter Basic 20 with measurement uncertainties of ±0.01 in pH and ±0.1 K in temperature. The used concentrations were chosen keeping in mind both the solubility of the antioxidants in water and the useful measurement range of the spectrophotometer. When required, pH was corrected by adding drops of a 0.5 M sodium hydroxide (NaOH) aqueous solution and mixing for 30 min in an IKA RO 10P magnetic stirrer. Then, the concentrations were recalculated considering the added quantity of the pH adjuster. Afterwards, a Thermo Scientific Varioskan Flash spectrophotometer with an uncertainty of ±10^−4^ was used to carry out a UV-Vis absorbance scanning from 200 to 600 nm, with temperature stabilisation of the 200 μL samples at 298.15 K. Lastly, to test the stability of the UV-Vis absorbance spectra, the solutions were left to settle for 3 days without any especial protection from daylight, and the absorbance scanning was repeated with the Varioskan Flash spectrophotometer following the same procedure. To guarantee homogeneity, the solutions were stirred for 30 min in the IKA RO 10P magnetic stirrer before the UV-Vis absorbance measurements. The concentrations were normalised to reduce the effect of dilution by the pH adjuster’s addition (NaOH) on the absorbance spectra using:

(4) $$A^{\prime }=A\cdot \frac{C_{\mathrm{pH}=7.5}}{C_{\mathrm{pH}=k}}$$

where A’ refers to the normalized absorbance, A is the experimental UV-Vis absorbance for a given wavelength (λ), $C_{\mathrm{pH}=7.5}$ is the reference concentration at pH = 7.5 and $C_{\mathrm{pH}=k}$ is the concentration of the stock solution of biomolecule at pH = k.

#### 2.2.2. UV-Vis Absorbance Calibration Curves

UV-Vis absorbance calibration curves were determined for gallic acid and ferulic acid at the conditions of the to-be-used extractive media (pH = 7.5, P = 0.1 MPa and T = 298.15 K) by measuring the UV-Vis absorbance of known concentrations of the antioxidants at the wavelength of local maxima (260 and 310 nm, respectively) using the Thermo Scientific Varioskan Flash spectrophotometer. These solutions were prepared by weighing the solutes and purified water in the ADAM AAA 250 L balance and pH was adjusted with a 0.5 M aqueous solution of NaOH. The pH measurements were performed with a Crison pH meter Basic 20. Next, the absorbances of the blanks (purified water and plate) were subtracted from the experimental values and a first-degree fitting was performed, obtaining the absorbance-concentration calibration curves, as Equation (5) shows.

(5) A=αC+β

where A is absorbance, α is the slope of the calibration curve (absorptivity), C is the antioxidant concentration (in g·mL^−1^) and β is the y-intercept.

#### 2.2.3. Liquid-Liquid Equilibria

In this work, the liquid-liquid equilibria (LLE), i.e., tie-line compositions and coexistence curves, of the used ternary systems were not determined due to their availability in a previous work of the research group [29]. Table 2 shows the tie-line compositions and tie-line lengths (TLL) for the applied Aqueous Two-Phase Systems (ATPS): {ethyl lactate (1) + trisodium citrate (2) + water (3)} and {ethyl lactate (1) + tripotassium citrate (2) + water (3)}.

#### 2.2.4. Extraction of Biomolecules

To study the migration of gallic acid (GA) and ferulic acid (FA) in the mentioned ATPS, six vials with 10 mL were prepared for each system with the mass composition of the reported tie-lines, shown in Table 2, by pipetting pure ethyl lactate, pure water and the aqueous solutions of trisodium citrate (30.34 *m*%) and tripotassium citrate (32.75 *m*%). In this process, 1 mL of water was replaced with 1 mL of stock solution of antioxidant (5.97 · 10^−4^ g/mL of GA or 2.60 · 10^−4^ g/mL of FA), measured with an Eppendorf Multipipette E3x electronic pipette, with a measurement uncertainty of 0.5 μL when using the 200 μL tips. The masses of all pipetted volumes were assessed with an ADAM AAA 250L balance. Afterwards, the vials were capped and sealed with parafilm to avoid moisture-content variations and were stirred in a VWR VV3 vortex for 2 min, after which they were left under stirring for 6 h in a Julabo F12 thermostatic bath at 298.15 K. Then, the vials were left to settle overnight, which corresponds to about 12 h, at 298.15 K and 0.1 MPa, and the liquid phases (top and bottom) were separated using pipettes. Moreover, the respective masses (m) were determined with an ADAM AAA 250 L balance, UV-Vis absorbances (A) with a Thermo Scientific Varioskan Flash spectrophotometer, pH values with a Crison pH meter Basic 20 and densities (ρ) with an Anton Paar DSA-5000M densimeter, with measurement uncertainties of $\pm 3\cdot 10^{5}$ g·cm^−3^ in density and ±0.01 K in temperature.

Then, to evaluate the performed phase separation, the mass losses were determined using:

(6) $$L_{m}=\frac{m_{2}-m_{1}}{m_{1}}\cdot 100$$

where $m_{1}$ is the feed mass and $m_{2}$ is the sum of masses of the two separated phases.

Afterwards, the volume of each phase was calculated using the assessed masses and densities, as Equation (7) shows.

(7) $$V_{i}^{f}=\frac{m_{i}^{f}}{\rho_{i}^{f}}$$

where i is the tie-line number, f refers to the top or bottom phase, V is the phase volume, m is the measured phase mass and ρ is the measured phase density.

Next, the antioxidant concentrations in each phase were obtained from the measured UV-Vis absorbance by the determined calibration curves in Section 2.2.2 (after having subtracted the corresponding blanks), and the partition coefficients (K) were calculated using:

(8) $$K_{i}=\frac{C_{i}^{\mathrm{top}}}{C_{i}^{\mathrm{bottom}}}$$

where i is the tie-line number and $C_{\;}^{\mathrm{top}}$ and $C_{\;}^{\mathrm{bottom}}$ refer to the antioxidant concentrations in the top and bottom phases, respectively.

To validate UV-Vis absorbance as an analytical method, the mass balance was checked for each tie-line by calculating the mass losses in quantification ($L_{s}$), as Equation (9) shows.

(9) $$L_{s,i}=\frac{m_{s2,i}-m_{s1,i}}{m_{s1,i}}\cdot 100$$

where $m_{s1}$ is the added mass of antioxidant (present in 1 mL of stock solution, as previously explained) and $m_{s2}$ is the quantified experimental mass of antioxidant, which was calculated following:

(10) $$m_{s2,i}=\sum C_{i}^{f}V_{i}^{f}$$

Finally, the extraction efficiencies (E) were calculated for each tie-line using Equation (11).

(11) $$E_{i}=\frac{m_{s2}}{m_{s1}}\cdot 100$$

## 3. Results and Discussion

### 3.1. Effect of pH in the Mean Electrical Charge

**Figure 1:**
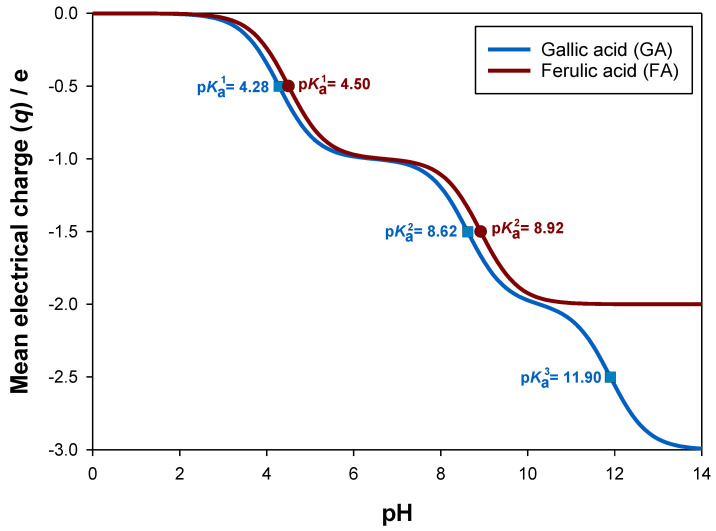
Calculated mean electrical charge (q) for gallic acid and ferulic acid, expressed in terms of the elementary charge (e), i.e., 1.602 · 10 ^−19^ C.

The correct assessment of the mean electrical charge (q) of biomolecules is vital to appropriately evaluate the stability, antioxidant activity and solubility of each differently charged species [48]. The electronic properties of gallic acid (GA) and ferulic acid (FA) are well-reported in literature, so their p*K*_a_ values (4.28, 8.62 and 11.90 [45] for GA, and 4.50 and 8.92 [46] for FA) are known. Therefore, using Equation (3), the mean electrical charge (q) of these biomolecules was calculated as function of pH, as Figure 1 shows. Since the lower p*K*_a_ values of gallic acid and ferulic acid are approximately equal, their mean electrical charges and distributions of charged species, i.e., relative abundance of each antioxidant stage, are alike up to pH = 10, as Tables S1 and S2, in the Supplementary Materials, show for GA and FA, respectively.

### 3.2. Effect of pH in the Absorbance Spectra

After having determined the mean electrical charges (q) as a function of pH for gallic acid and ferulic acid, the effect of pH on the UV-Vis absorbance spectra was assessed by preparing concentrations of each antioxidant at different pH values and measuring absorbance, as explained in Section 2.2.1.

**Figure 2:**
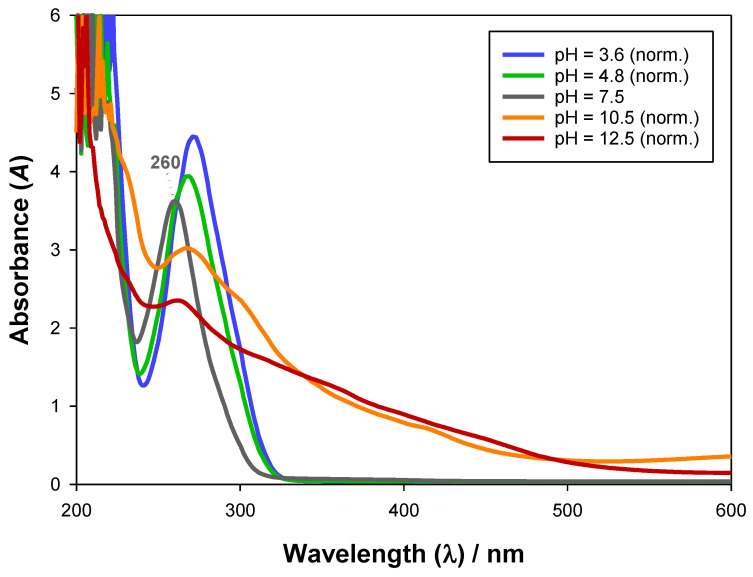
Influence of pH on the UV-Vis absorbance spectra of gallic acid (~1.5·10^−4^ g·mL^−1^) at 298.15 K and 0.1 MPa.

In Figure 2, the UV-Vis absorbance spectra of gallic acid can be observed. A significant variation of the spectra with pH was noted, particularly concerning the 250–300 nm range, in which a general decrease in absorbance with growing pH and a relative maximum shift (260 nm, pH = 7.5) occurred. These alterations in the UV-Vis absorbance spectra, besides being caused by the known protolytic equilibrium (proton transfer), are also thought to be due to changes in chemical conformation (e.g., orbital transitions) [49]. Furthermore, at larger pH values (10.5 and 12.5), the UV-Vis absorbance spectra followed a completely different behaviour, which was observed to be irreversible, hinting that a chemical reaction such as oxidation of gallic acid may have been taking place [49]. Moreover, as Figures S1–S5 in the Supplementary Materials show, conversely to what was found at pH = 3.6, 4.8 and 7.5, the absorbance spectra drastically changed after 3 days for pH = 10.5 and 12.5, reinforcing the hypothesis of a chemical reaction at high pH values [50].

**Figure 3:**
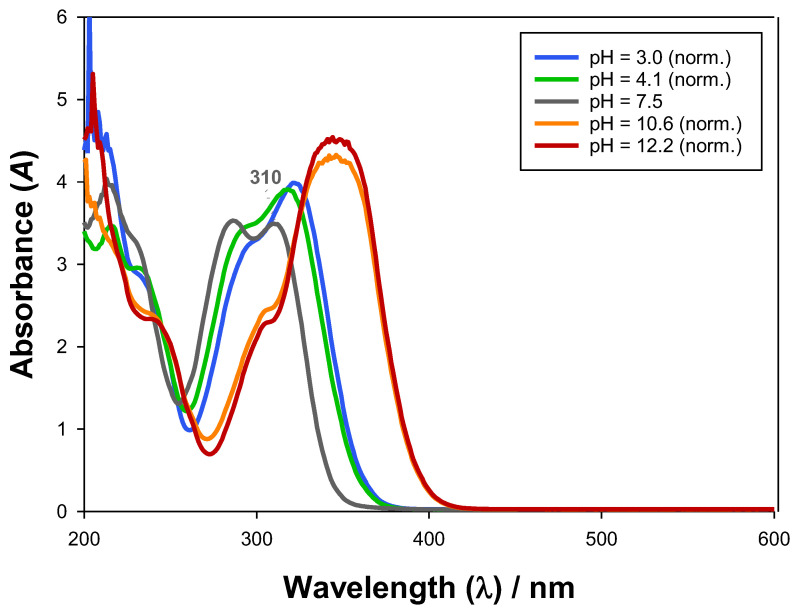
Influence of pH in the UV-Vis absorbance spectra of ferulic acid (~8.0·10^−5^ g·mL^−1^) at 298.15 K and 0.1 MPa.

The effect of pH on the UV-Vis absorbance spectra of ferulic acid was also delved into, as Figure 3 shows. Ferulic acid also presented a general absorbance decrease with larger pH and a shift in the relative maximum (310 nm, pH = 7.5) in the 250–300 nm range due to the protolytic equilibrium. Moreover, atypical absorbance spectra were observed for large pH values (pH = 10.6 and 12.2) as well, but they were found to be reversible and stable with time, as Figures S6–S10 in the Supplementary Materials show. Thus, ferulic acid was considered stable at all tested pH values, as Table 3 shows, and the alterations in the UV-Vis absorbance spectra were justified by the protolytic equilibrium and by reversible changes in chemical modification.

As Table 3 shows, both gallic acid and ferulic acid exhibited stable and reversible UV-Vis absorbance spectra at pH = 7.5, which is close to the reported pH of the studied ATPS (as seen in Section 2.2.3). Being so, the application of these ternary systems to extract GA and FA was validated and UV-Vis absorbance calibration curves were determined at 298.15 K and 0.1 MPa, as Figure S11 in the Supplementary Materials illustrates. The calibration curves were carried out with aqueous solutions of antioxidant prepared roughly one day earlier to correspond to the period composed by mixing and settling required for the partition studies.

### 3.3. Partition Coefficients and Extraction Efficiencies

After having prepared feed mixtures corresponding to the known tie-line compositions, the samples were left stirring for 6 h and settling overnight (~12 h), as described in Section 2.2.4. After equilibrium was reached, the top and bottom phases were separately removed using pipettes and the phases were characterised by measuring mass, UV-Vis absorbance, pH and density, as Table 4 shows. Absorbances were converted to mass concentrations using the predetermined calibration curves.

As can be seen in Table 4, small mass losses were observed in phase separation. As expected, bottom phases were significantly denser than top phases and the obtained pH were close to 7.5. Further, bottom phases presented lower antioxidant concentrations, which hints that the biomolecules preferentially diffused into the top phases. This fact was confirmed by the calculated performance indicators: partition coefficients (K) higher than unity and extraction efficiencies (E) higher than 50 %, as Table 5 shows.

**Figure 4:**
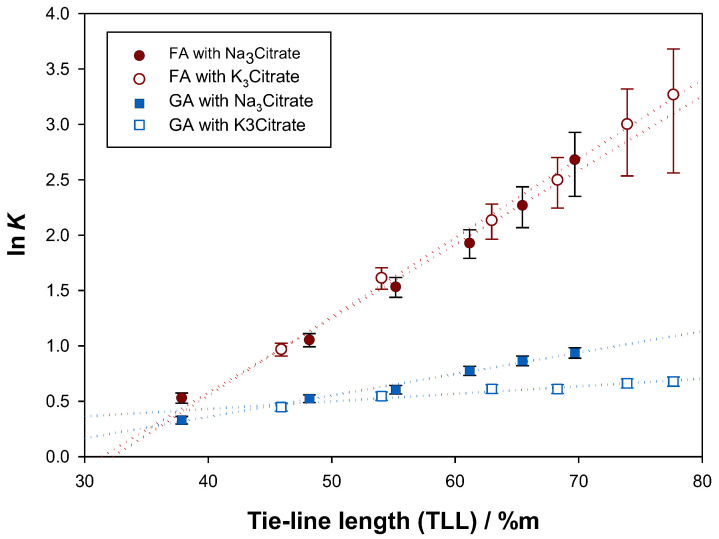
Relation between the natural logarithms of the experimental partition coefficients (K) and the tie-line lengths (TLL) for gallic acid (GA) and ferulic acid (FA) in the ATPS {ethyl lactate (1) + trisodium citrate (2) + water (3)} and {ethyl lactate (1) + tripotassium citrate (2) + water (3)} at 298.15 K and 0.1 MPa.

In Figure 4, the natural logarithm of the calculated partition coefficients (K) was represented as a function of tie-line length (TLL). The largest partition coefficients were obtained for ferulic acid, so it can be concluded that the top phases of the studied ternary systems presented more favourable hydrophobicity and bio-specific affinity towards this antioxidant [51]. This may be caused by a more beneficial size, polarity, chemical conformation and mean electrical charge of ferulic acid compared to gallic acid [51,52]. Moreover, the positive slope of the first-degree fittings hints that top phase-oriented solute migration is favoured both for ferulic acid and gallic acid with larger tie-line lengths, i.e., with more distinct phase compositions (top phases richer in ethyl lactate and bottom phases richer in organic salt), which will be useful for the scale-up of the studied extractions.

**Figure 5:**
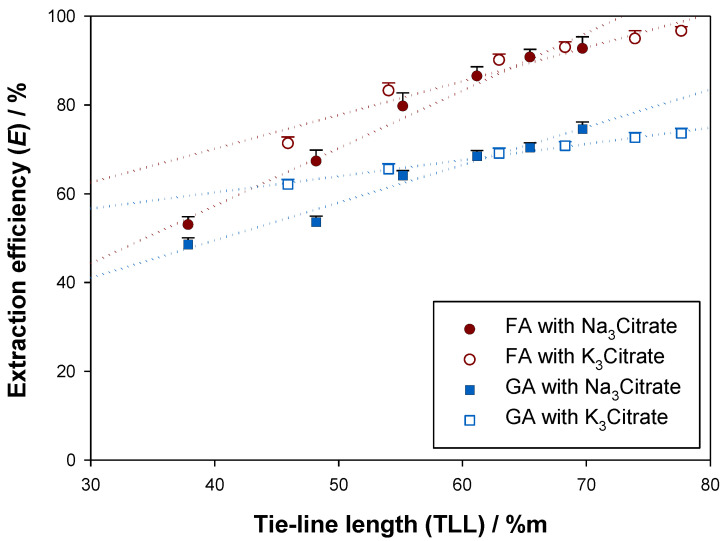
Relation between the extraction efficiencies (E) and the tie-line lengths (TLL) for gallic acid (GA) and ferulic acid (FA) in the ATPS {ethyl lactate (1) + trisodium citrate (2) + water (3)} and {ethyl lactate (1) + tripotassium citrate (2) + water (3)} at 298.15 K and 0.1 MPa.

Concerning extraction efficiencies (*E*), ferulic acid obtained maximum values close to 100 %, while gallic acid never surpassed 80 %, as Figure 5 shows. Conversely to what was observed for the partition coefficients (K), the slopes of the first-degree fittings of the extractive efficiencies with the tie-line lengths (TLL) seem to be ruled by the ternary system being used rather than by the biomolecule being extracted, since Na_3_Citrate-containing ATPS (and K_3_Citrate-containing ATPS) yielded similar slopes. Regarding the performance of the salting-out agents, i.e., species which promote ethyl lactate-water immiscibility, no significant difference was found in the use of Na_3_Citrate or K_3_Citrate for longer tie-line lengths considering that the performance indicators (K and E) were mostly alike.

### 3.4. Effect of Tie-Line Composition in the Antioxidant Stages Distribution

**Figure 6:**
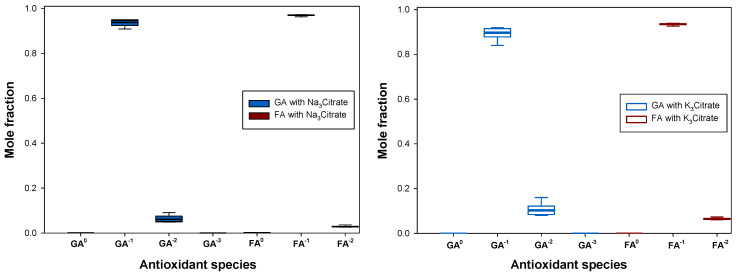
Effect of the tie-line compositions in the calculated mole fractions of the antioxidant stages of gallic acid (GA) and ferulic acid (FA) in the ATPS {ethyl lactate (1) + trisodium citrate (***left***) or tripotassium citrate (***right***) (2) + water (3)} at 298.15 K and 0.1 MPa. GA^0^, GA^−1^, GA^−2^ and GA^−3^ refer to the antioxidant stages of gallic acid with electrical charges equal to 0, −1, −2, −3 and −4 e, respectively; FA^0^, FA^−1^ and FA^−2^ refer to the antioxidant stages of ferulic acid with electrical charges equal to 0, −1 and −2 e, respectively, and e stands for the elementary charge (1.602∙10^−19^ C).

Depending on the composition of each tie-line, different pH values may be obtained in the phases, which may lead to different distributions of antioxidant stages when p*K*_a_ values are near the pH of the system. Determining whether the tested tie-line compositions extract the same antioxidant stages is particularly important when one of the antioxidant stages is too reactive (chemically unstable), less effective than the others (smaller antioxidant activity) or undetectable by the analytical method (e.g., presents no UV-Vis absorbance). The variations in the mean electrical charge (q) of gallic acid and ferulic acid in the tie-lines were determined and can be seen in Figure 6, which provides an estimation for the relative abundance of the antioxidant stages based on the measured pH and known p*K*_a_ values. More precise experimental determinations regarding the exact relative abundance of each species and/or separation of differently charged species may be accomplished by other techniques such as chromatography. It was concluded that the tested tie-line compositions yielded the same distribution of electrical charges for ferulic acid. However, for gallic acid, some small variations were observed in the abundance of the species with q=−1 and q=−2 e. Since no enhanced reactivity or instability was noted at such pH values (7–8), these variations were considered negligible.

Thus, having in mind the favourable performance indicators (K and E) obtained, the extraction of gallic acid and ferulic acid in the longer tie-lines of these green ATPS was considered promising for larger scale operation, and the solutes were considered sufficiently well-characterised for the future evaporation of the solvent in a vacuum rotary evaporator at 318.15 K. This will allow to produce a solid rich in antioxidants (gallic acid and ferulic acid) and in organic salts commonly found in food products (Na_3_Citrate and K_3_Citrate) for future application in dietary supplements with hair fortification purposes.

## 4. Conclusions

In this work, partition studies of gallic acid (GA) and ferulic acid (FA) were successfully performed in the Aqueous Two-Phase Systems (ATPS) {ethyl lactate (1) + trisodium citrate or tripotassium citrate (2) + water (3)} at 298.15 K and 0.1 MPa for future application of these biomolecules in food supplements for hair fortification. The studied ATPS provided biocompatible and sustainable media for the extraction of GA and FA, with the latter obtaining the best results: maximum partition coefficients (*K*) of 15 ± 5 and (3 ± 2) · 10^1^ and maximum extraction efficiencies (*E*) of (92.7 ± 0.4)% and (96.7 ± 0.4)% for the longest tie-lines (TLL = 69.68 and 77.66 *m*%) in {ethyl lactate (1) + trisodium citrate (2) + water (3)} and {ethyl lactate (1) + tripotassium citrate (2) + water (3)}, respectively. On the other hand, gallic acid only obtained maximum partition coefficients (*K*) of 2.6 ± 0.2 and 1.97 ± 0.09 and maximum extraction efficiencies (*E*) of (76.2 ± 0.2)% and (74.7 ± 0.2)% for the longest tie-lines (TLL = 69.68 and 77.66 *m*%) in {ethyl lactate (1) + trisodium citrate (2) + water (3)} and {ethyl lactate (1) + tripotassium citrate (2) + water (3)}, respectively. Moreover, the effect of pH on the UV-Vis absorbance spectra was evaluated for all biomolecules to minimise errors in solute quantification (< 3 %) and validate the determined partition coefficients and extraction efficiencies. Both GA and FA were found to be stable at the used extractive conditions, with only protolytic equilibrium or reversible changes in chemical conformation taking place, for which their extraction using the studied ATPS could provide an eco-friendly method to produce antioxidant-rich dietary supplements to tackle hair loss.

## Figures and Tables

**Table 1:** List of chemicals used in this work, with respective chemical formula, supplier, purity, CAS number and abbreviation.

Chemical	Supplier	Purity/*m*% ^a,b^	CAS	Abbreviation
Ethanol (CH_3_CH_2_OH)	Sigma-Aldrich	>99	64-17-5	EtOH
(-)-ethyl L-lactate (C_5_H_10_O_3_)	Sigma-Aldrich	>98	97-64-3	EL
Ferulic acid (C_10_H_10_O_4_)	Sigma-Aldrich	>99	537-98-4	FA
Gallic acid (C_7_H_6_O_5_)	Fluka	>98	149-91-7	GA
Potassium citrate monohydrate (C_6_H_5_K_3_O_7_·H_2_O)	Sigma-Aldrich	>99	6100-05-6	K_3_Citrate
Purified water (H_2_O)	VWR chemicals	-	7732-18-5	W
Sodium hydroxide (NaOH)	Merck	>99	1310-73-2	NaOH
Sodium citrate tribasic dihydrate (C_6_H_5_Na_3_O_7_·2H_2_O)	Sigma-Aldrich	>99	6132-04-3	Na_3_Citrate

^a^ Provided by the supplier. ^b^ *m*% refers to mass percentage.

**Table 2:** Determined tie-lines for the ATPS {ethyl lactate (1) + trisodium citrate or tripotassium citrate (2) + water (3)} at 298.15 K and 0.1 MPa ^a,b^ [29].

Tie-Line	Feed		TLL/*m*%	Phase	Separation		
	*w*_1_/*m*%	*W*_2_/*m*%			*w*_1_/*m*%	*w*_2_/*m*%	pH
**{EL (1) + Na_3_Citrate (2) + water (3)}**							
**1**	30.0	11.0	37.85	Top	51.7	3.0	7.00
				Bottom	16.0	15.7	6.98
**2**	32.0	11.4	48.18	Top	57.5	2.0	6.98
				Bottom	12.3	18.5	6.96
**3**	34.3	11.7	55.17	Top	61.5	1.4	6.98
				Bottom	9.8	20.7	6.97
**4**	36.5	12.1	61.17	Top	65.0	1.0	7.00
				Bottom	7.9	23.0	7.00
**5**	38.5	12.3	65.44	Top	67.7	0.7	6.98
				Bottom	6.8	24.7	6.97
**6**	40.6	12.6	69.68	Top	70.1	0.5	6.98
				Bottom	5.5	26.6	7.00
**{EL (1) + K_3_Citrate (2) + water (3)}**							
**1**	35.5	12.6	45.91	Top	57.9	3.9	7.21
				Bottom	15.0	20.3	7.39
**2**	37.5	13.0	54.02	Top	61.7	3.4	7.22
				Bottom	11.5	23.2	7.41
**3**	39.2	13.5	62.96	Top	67.4	2.1	7.19
				Bottom	9.1	25.8	7.37
**4**	41.1	13.9	68.28	Top	70.4	1.6	7.23
				Bottom	7.5	28.2	7.41
**5**	43.0	14.3	73.92	Top	73.6	1.1	7.12
				Bottom	6.0	31.0	7.39
**6**	44.6	14.8	77.66	Top	75.8	0.9	7.22
				Bottom	5.2	33.1	7.43

^a^ ***w*_1_** stands for the mass percentage (*m*%) of species i. ^b^ Standard uncertainties (*u*) are: *u*(*T*) = 0.2 K, *u*(*P*) = 10 kPa, *u*(*w_i_*) = 10^−1^ and *u*(pH) = 10^−2^.

**Table 3:** Stability of the UV-Vis absorbance spectra of gallic acid (GA) and ferulic acid (FA) after settling for 3 days at 298.15 K and 0.1 MPa.

Biomolecule	pH = 3	pH = 4	pH = 5	pH = 7.5	pH = 11	pH = 12	pH = 13
**Gallic acid**	-	Stable	Stable	Mostly stable	Unstable ^NR^	-	Unstable ^NR^
**Ferulic acid**	Stable	Stable	-	Stable	Stable	Stable	-

**^N^**^R^ marks non-reversible changes in the UV-Vis absorbance spectra.

**Table 4:** Experimental phase mass (*m*), biomolecule mass losses (***L*_m_**), biomolecule concentration (*C*), phase density (*ρ*) and phase pH for the top and bottom phases in the extraction of gallic acid or ferulic acid in the ATPS {ethyl lactate (1) + trisodium citrate or tripotassium citrate (2) + water (3)} at 298.15 K and 0.1 MPa ^a^.

Tie-Line	Phase	*m*/g	*L*_m_/%	*C*/g·mL^−1^	*ρ*/g·mL^−1^	pH
		**Gallic Acid in {EL (1) + Na_3_Citrate (2) + water (3)}**				
**1**	Top	3.9712	−0.52	8.11 · 10^−5^	1.05717	7.36
	Bottom	6.0217		5.83 · 10^−5^	1.12267	7.33
**2**	Top	3.9556	−0.25	8.90 · 10^−5^	1.04894	7.33
	Bottom	6.0885		5.27 · 10^−5^	1.13875	7.33
**3**	Top	4.7830	−0.69	8.77 · 10^−5^	1.04395	7.35
	Bottom	5.2566		4.79 · 10^−5^	1.15652	7.36
**4**	Top	4.7938	−1.12	9.34 · 10^−5^	1.04385	7.36
	Bottom	5.1479		4.30 · 10^−5^	1.17005	7.56
**5**	Top	4.8096	−0.66	9.56 · 10^−5^	1.04291	7.39
	Bottom	5.2244		4.02 · 10^−5^	1.17719	7.62
**6**	Top	5.1917	−0.49	9.38 · 10^−5^	1.04110	7.44
	Bottom	4.8698		3.67 · 10^−5^	1.19781	7.62
		**Ferulic acid in {EL (1) + Na_3_Citrate (2) + water (3)}**				
**1**	Top	4.0326	−0.41	3.82 · 10^−5^	1.06631	7.49
	Bottom	5.9923		2.25 · 10^−5^	1.09589	7.50
**2**	Top	4.2842	−0.20	4.56 · 10^−5^	1.06154	7.36
	Bottom	5.7625		1.59 · 10^−5^	1.11329	7.37
**3**	Top	4.8229	−0.50	4.74 · 10^−5^	1.05130	7.34
	Bottom	5.2068		1.02 · 10^−5^	1.13350	7.39
**4**	Top	5.0262	−0.49	4.92 · 10^−5^	1.04683	7.34
	Bottom	4.9886		7.15 · 10^−6^	1.14866	7.36
**5**	Top	5.3019	−0.36	4.84 · 10^−5^	1.04361	7.38
	Bottom	4.6933		5.00 · 10^−6^	1.16247	7.35
**6**	Top	5.5474	−0.21	4.75 · 10^−5^	1.04404	7.42
	Bottom	4.5557		3.26 · 10^−6^	1.17527	7.36
		**Gallic acid in {EL (1) + K_3_Citrate (2) + water (3)}**				
**1**	Top	5.0341	−0.67	8.29 · 10^−5^	1.06598	7.56
	Bottom	4.9839		5.27 · 10^−5^	1.13952	7.68
**2**	Top	5.1521	−0.51	8.58 · 10^−5^	1.06438	7.56
	Bottom	4.8572		4.94 · 10^−5^	1.14674	7.70
**3**	Top	5.3737	−0.42	8.58 · 10^−5^	1.05704	7.58
	Bottom	4.6898		4.63 · 10^−5^	1.16473	7.70
**4**	Top	5.4942	−0.40	8.54 · 10^−5^	1.05212	7.59
	Bottom	4.5353		4.61 · 10^−5^	1.18149	7.82
**5**	Top	5.5606	−0.50	8.65 · 10^−5^	1.04930	7.61
	Bottom	4.4468		4.44 · 10^−5^	1.19981	7.78
**6**	Top	5.5914	−0.70	8.61 · 10^−5^	1.04942	7.65
	Bottom	4.3850		4.35 · 10^−5^	1.21668	7.93
		**Ferulic acid in {EL (1) + K_3_Citrate (2) + water (3)}**				
**1**	Top	4.8465	−0.52	8.29 · 10^−5^	1.07341	7.75
	Bottom	5.1568		5.27 · 10^−5^	1.13804	7.73
**2**	Top	5.0310	−0.34	8.58 · 10^−5^	1.05944	7.75
	Bottom	4.9931		4.94 · 10^−5^	1.16013	7.74
**3**	Top	5.2857	−0.40	8.58 · 10^−5^	1.05433	7.73
	Bottom	4.7126		4.63 · 10^−5^	1.17877	7.76
**4**	Top	5.3688	−0.47	8.54 · 10^−5^	1.04734	7.75
	Bottom	4.6258		4.61 · 10^−5^	1.19250	7.78
**5**	Top	5.5171	−0.67	8.65 · 10^−5^	1.04685	7.75
	Bottom	4.4224		4.44 · 10^−5^	1.21242	7.81
**6**	Top	5.7985	−0.61	8.61 · 10^−5^	1.04685	7.75
	Bottom	4.3484		4.35 · 10^−5^	1.21885	7.82

^a^ The measurement uncertainties (*u*) are: *u*(*m*) = 10^−4^ g, *u*(*A*) = 10^−4^, *u*(*ρ*) = 3 · 10^−5^ g mL^−1^ and *u*(pH) = 10^−2^.

**Table 5:** Calculated solute losses (***L*_S_**), extraction efficiency (*E*) intervals, partition coefficients (*K*) and literature-based tie-line lengths (TLL) for the extraction of gallic acid and ferulic acid in the ATPS {ethyl lactate (1) trisodium citrate or tripotassium citrate (2) + water (3)} at 298.15 K and 0.1 MPa.

Tie-Line	*L*_S_/%	*E*/%	*K*	TLL/*m*%
**Gallic acid in {EL (1) + Na_3_Citrate (2) + water (3)}**				
**1**	−1.47	(48.62–50.08) ± 0.07	1.39 ± 0.05	37.85
**2**	−1.32	(53.64–54.97) ± 0.08	1.69 ± 0.06	48.18
**3**	−1.05	(64.16–65.21) ± 0.09	1.83 ± 0.07	55.17
**4**	−1.28	(68.5–69.2) ± 0.1	2.17 ± 0.09	61.17
**5**	−1.06	(70.4–71.5) ± 0.1	2.4 ± 0.2	65.44
**6**	−1.62	(74.6–76.2) ± 0.2	2.6 ± 0.2	69.68
**Ferulic acid in {EL (1) + Na_3_Citrate (2) + water (3)}**				
**1**	−1.79	(53.0–54.8) ± 0.3	1.70 ± 0.08	37.85
**2**	−2.50	(67.4–69.9) ± 0.3	2.9 ± 0.2	48.18
**3**	−2.98	(79.7–82.7) ± 0.4	4.6 ± 0.5	55.17
**4**	−2.09	(86.5–88.6) ± 0.4	6.9 ± 0.9	61.17
**5**	−1.77	(90.8–92.5) ± 0.4	10 ± 2	65.44
**6**	−2.63	(92.7–95.4) ± 0.4	15 ± 5	69.68
**Gallic acid in {EL (1) + K_3_Citrate (2) + water (3)}**				
**1**	−1.15	(62.10–63.25) ± 0.09	1.56 ± 0.06	45.91
**2**	−1.21	(65.57–66.77) ± 0.09	1.73 ± 0.07	54.02
**3**	−1.16	(69.1–70.3) ± 0.1	1.84 ± 0.08	62.96
**4**	−0.90	(70.8–71.7) ± 0.1	1.85 ± 0.08	68.28
**5**	−1.15	(72.7–73.8) ± 0.1	1.94 ± 0.08	73.92
**6**	−1.10	(73.6–74.7) ± 0.2	1.97 ± 0.09	77.66
**Ferulic acid in {EL (1) + K_3_Citrate (2) + water (3)}**				
**1**	−1.43	(71.4–72.8) ± 0.3	2.6 ± 0.2	45.91
**2**	−1.71	(83.2–85.0) ± 0.4	5.0 ± 0.5	54.02
**3**	−1.38	(90.1–91.5) ± 0.4	8 ± 2	62.96
**4**	−1.21	(93.0–94.2) ± 0.4	12 ± 3	68.28
**5**	−1.77	(95.0–96.7) ± 0.4	20 ± 8	73.92
**6**	−0.94	(96.7–97.6) ± 0.4	(3 ± 2) · 10^1^	77.66

## Data Availability

The data presented in this study are available on request from the corresponding author.

## Supplementary Materials

The following supporting information can be downloaded at: https://www.mdpi.com/article/10.3390/molecules28052369/s1, Figure S1: UV-Vis absorbance spectra of the aqueous stock solution of gallic acid (pH = 3.59) in the moment of preparation and after 3 days of settling at 298.15 K and 0.1 MPa; Figure S2: UV-Vis absorbance spectra of the aqueous stock solution of gallic acid (pH = 4.78) in the moment of preparation and after 3 days of settling at 298.15 K and 0.1 MPa; Figure S3: UV-Vis absorbance spectra of the aqueous stock solution of gallic acid (pH = 7.53) in the moment of preparation and after 3 days of settling at 298.15 K and 0.1 MPa; Figure S4: UV-Vis absorbance spectra of the aqueous stock solution of gallic acid (pH = 10.52) in the moment of preparation and after 3 days of settling at 298.15 K and 0.1 MPa; Figure S5: UV-Vis absorbance spectra of the aqueous stock solution of gallic acid (pH = 12.5) in the moment of preparation and after 3 days of settling at 298.15 K and 0.1 MPa; Figure S6: UV-Vis absorbance spectra of the aqueous stock solution of ferulic acid (pH = 3.0) in the moment of preparation and after 3 days of settling at 298.15 K and 0.1 MPa; Figure S7: UV-Vis absorbance spectra of the aqueous stock solution of ferulic acid (pH = 4.1) in the moment of preparation and after 3 days of settling at 298.15 K and 0.1 MPa; Figure S8: UV-Vis absorbance spectra of the aqueous stock solution of ferulic acid (pH = 7.5) in the moment of preparation and after 3 days of settling at 298.15 K and 0.1 MPa; Figure S9: UV-Vis absorbance spectra of the aqueous stock solution of ferulic acid (pH = 10.6) in the moment of preparation and after 3 days of settling at 298.15 K and 0.1 MPa; Figure S10: UV-Vis absorbance spectra of the aqueous stock solution of ferulic acid (pH = 12.2) in the moment of preparation and after 3 days of settling at 298.15 K and 0.1 MPa; Figure S11: UV-Vis absorbance calibration curves for gallic acid (λ = 260 nm) and ferulic acid (λ = 310 nm) at pH = 7.5, *T* = 298.15 K and *P* = 0.1 MPa; Table S1: Calculated mole fractions of each antioxidant stage and mean electrical charge (*q*) at different pH values for gallic acid (GA); Table S2: Calculated mole fractions of each antioxidant stage and mean electrical charge (*q*) at different pH values for ferulic acid (FA).

## Sample Availability

Samples of the compounds are not available from the authors.
