# Supplementary material for: Extraction of Gallic Acid and Ferulic Acid for Application in Hair Supplements
Source: Molecules. 2023 Mar 4;28(5):2369. doi: 10.3390/molecules28052369 (PMC10005415; doi:10.3390/molecules28052369)
Supplement: Supplementary file 1 [file molecules-28-02369-s001.zip › molecules-2236585-supplementary.pdf]

Supplementary Materials

Extraction of gallic acid and ferulic acid for application in hair supplements

Pedro Velho [1,2,a], Catarina S. Rebelo [1,2,b], Eugénia A. Macedo [1,2,*]

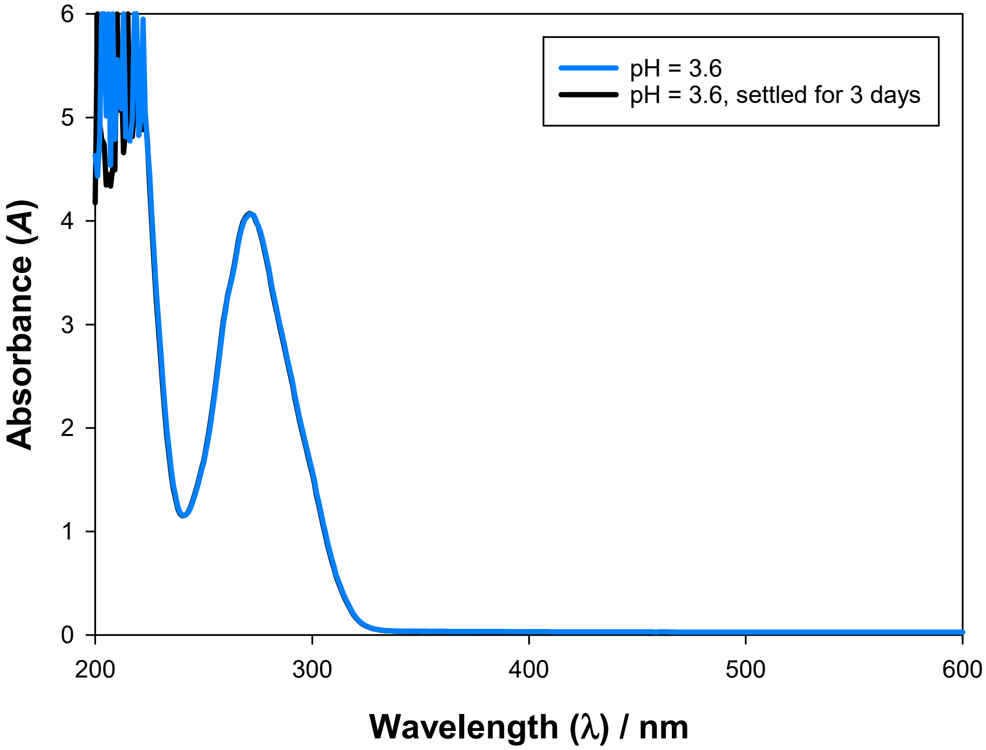

Figure S1. UV-Vis absorbance spectra of the aqueous stock solution of gallic acid ($1.36 \cdot 10^{-4}$ g·mL^{-1} and pH = 3.59) in the moment of preparation and after 3 days of settling at 298.15 K and 0.1 MPa.

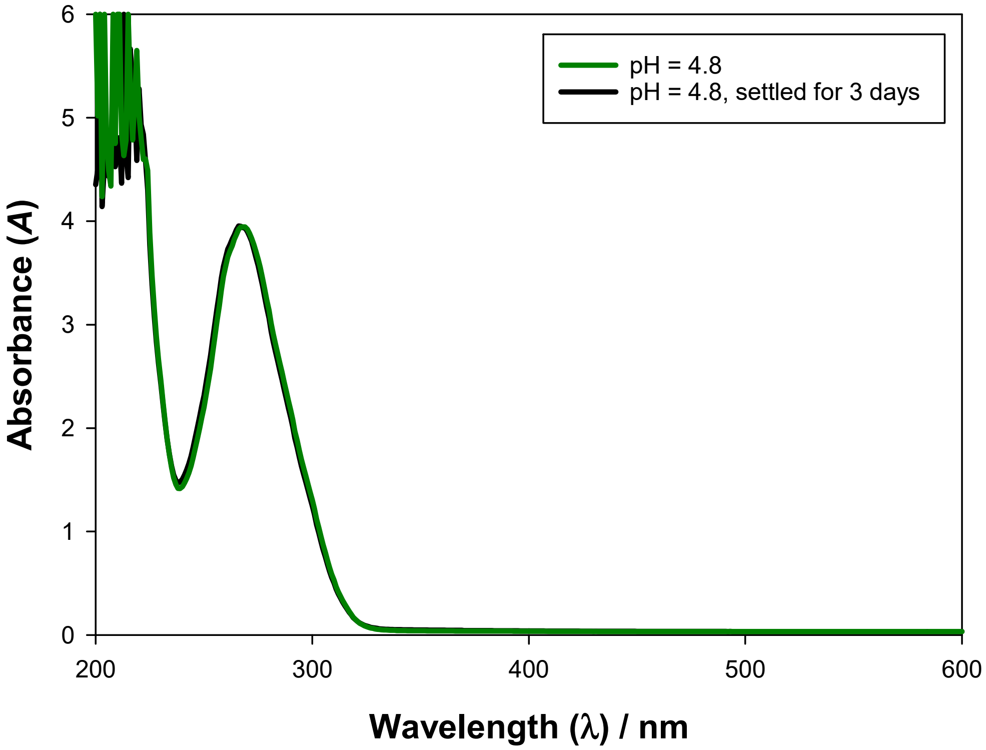

Figure S2. UV-Vis absorbance spectra of the aqueous stock solution of gallic acid ($1.49 \cdot 10^{-4}$ g·mL^{-1} and pH = 4.78) in the moment of preparation and after 3 days of settling at 298.15 K and 0.1 MPa.

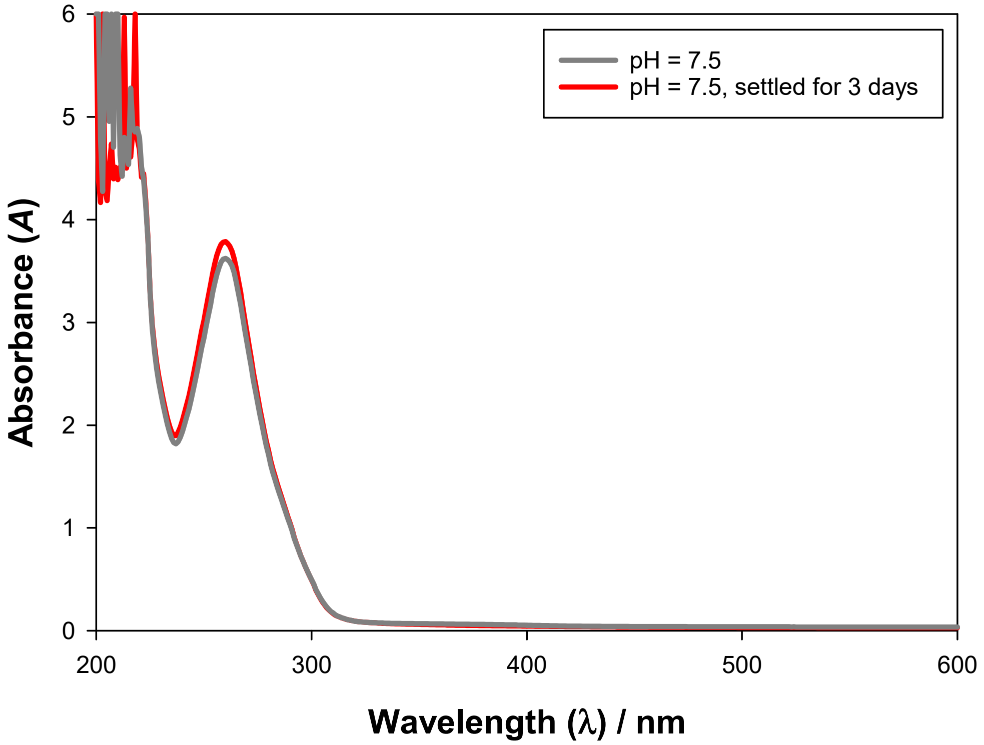

Figure S3. UV-Vis absorbance spectra of the aqueous stock solution of gallic acid ($1.49 \cdot 10^{-4}$ g·mL^{-1} and pH = 7.53) in the moment of preparation and after 3 days of settling at 298.15 K and 0.1 MPa.

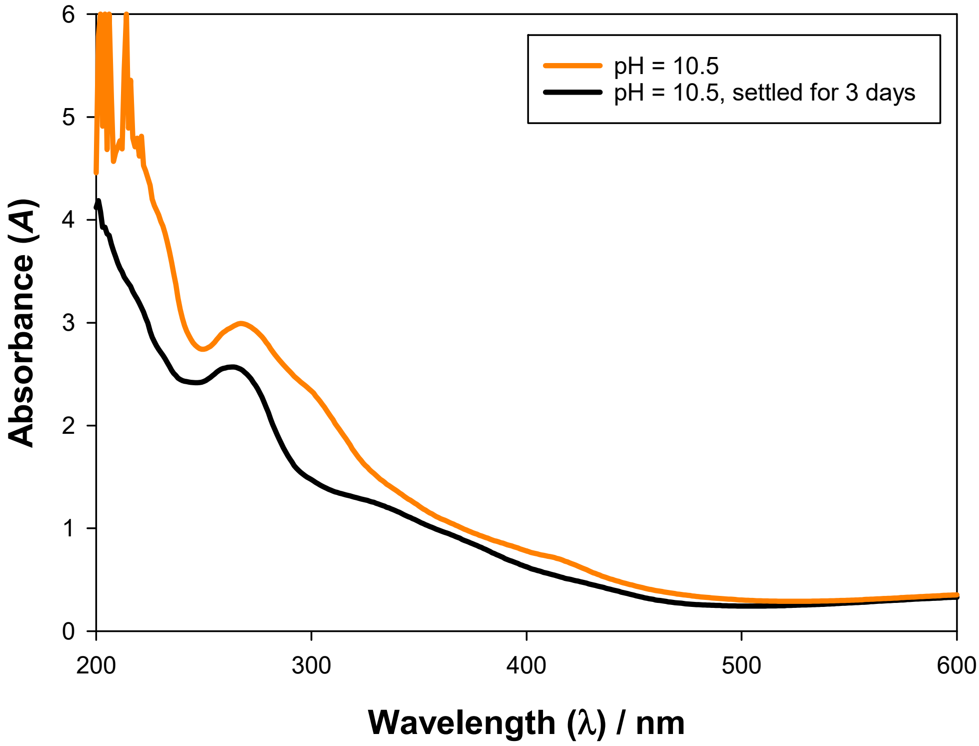

Figure S4. UV-Vis absorbance spectra of the aqueous stock solution of gallic acid ($1.48 \cdot 10^{-4}$ $g \cdot mL^{-1}$ and pH = 10.52) in the moment of preparation and after 3 days of settling at 298.15 K and 0.1 MPa.

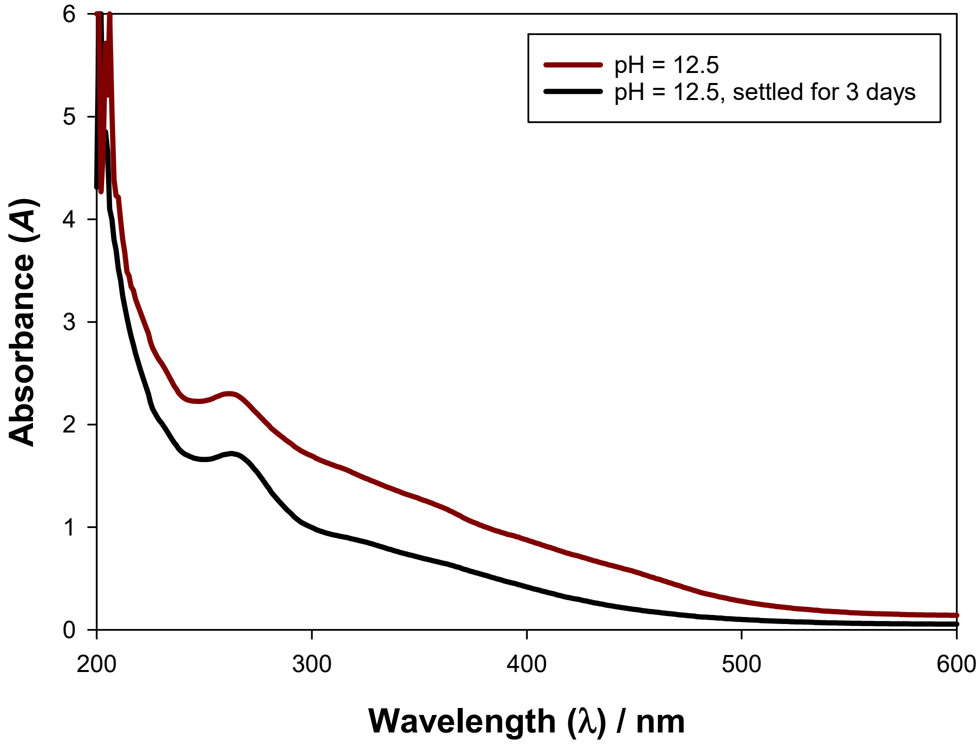

Figure S5. UV-Vis absorbance spectra of the aqueous stock solution of gallic acid ($1.46 \cdot 10^{-4}$ $g \cdot mL^{-1}$ and pH = 12.5) in the moment of preparation and after 3 days of settling at 298.15 K and 0.1 MPa.

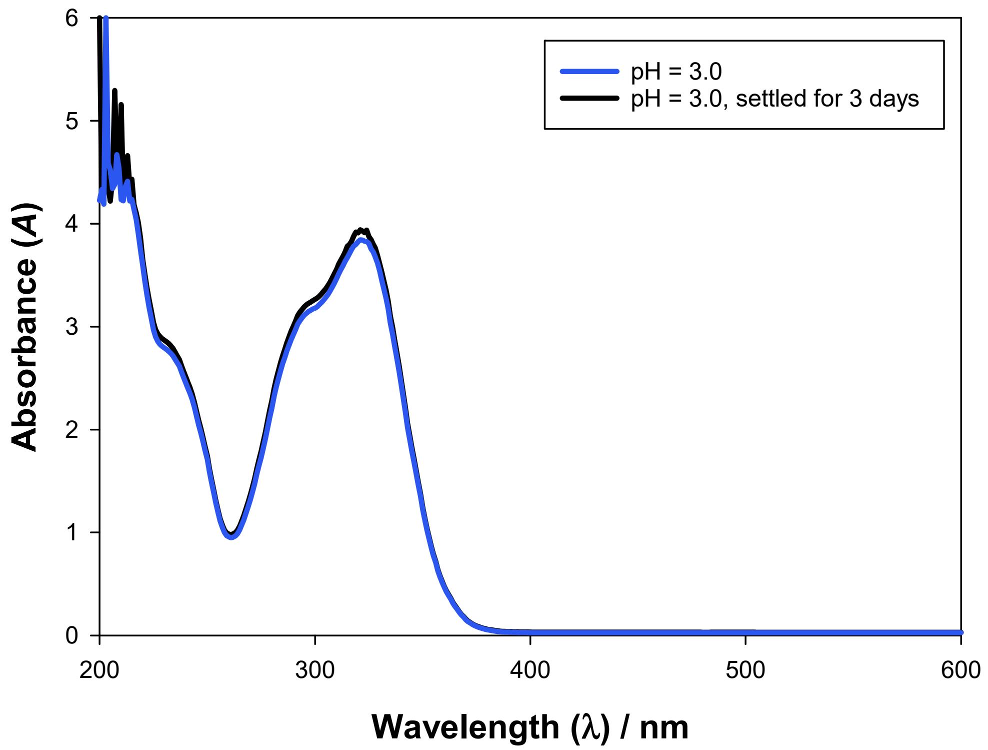

Figure S6. UV-Vis absorbance spectra of the aqueous stock solution of ferulic acid ($7.91{\cdot}10^{-5}$ g·mL^{-1} and pH = 3.0) in the moment of preparation and after 3 days of settling at 298.15 K and 0.1 MPa.

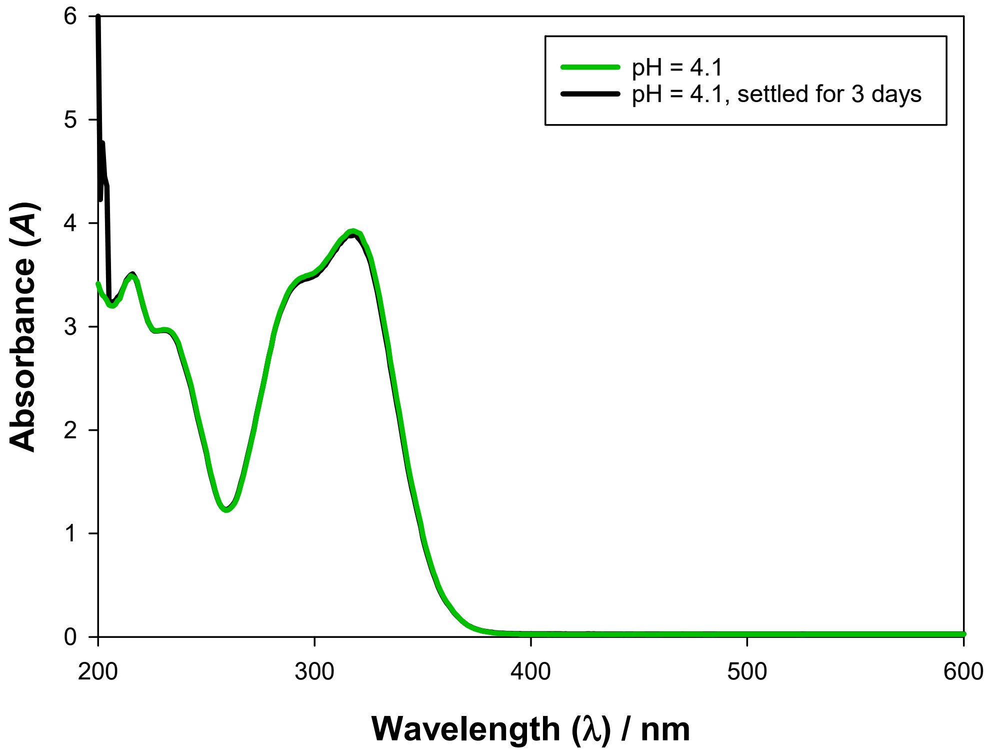

Figure S7. UV-Vis absorbance spectra of the aqueous stock solution of ferulic acid ($8.25{\cdot}10^{-5}$ g·mL^{-1} and pH = 4.1) in the moment of preparation and after 3 days of settling at 298.15 K and 0.1 MPa.

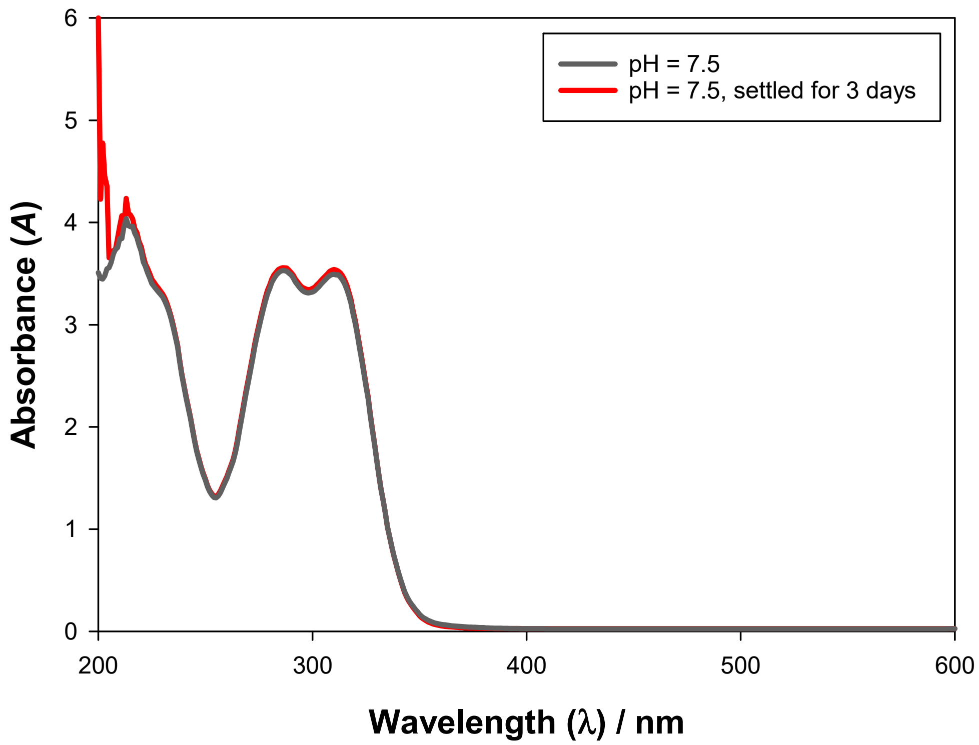

Figure S8. UV-Vis absorbance spectra of the aqueous stock solution of ferulic acid ($8.21 \cdot 10^{-5}$ g·mL^{-1} and pH = 7.5) in the moment of preparation and after 3 days of settling at 298.15 K and 0.1 MPa.

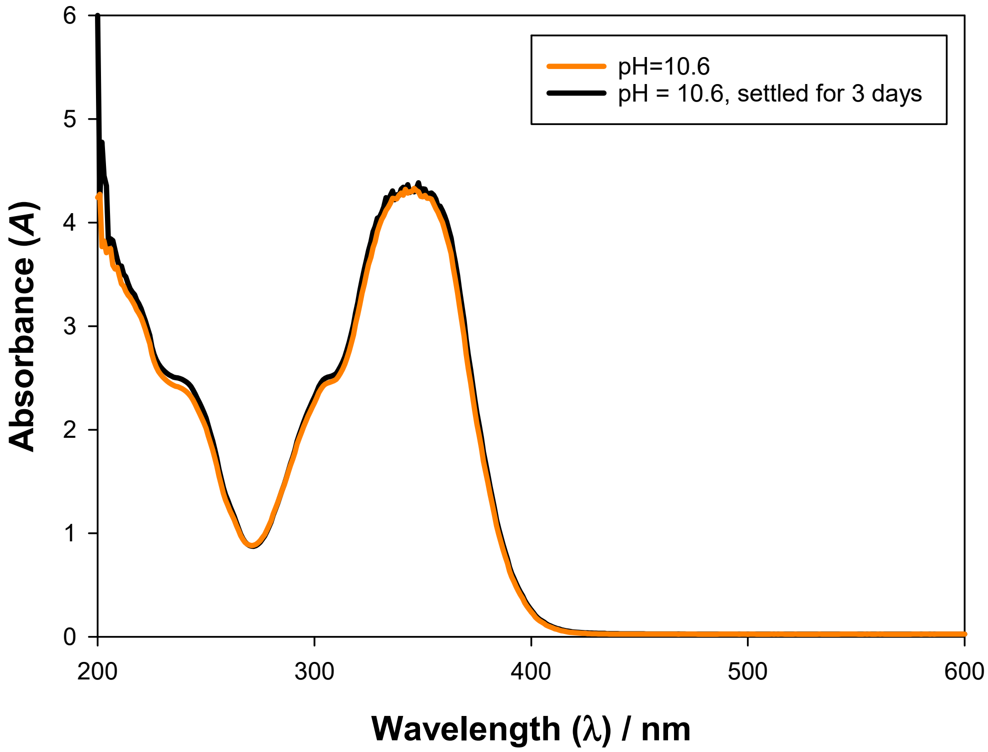

Figure S9. UV-Vis absorbance spectra of the aqueous stock solution of ferulic acid ($8.22 \cdot 10^{-5}$ g·mL^{-1} and pH = 10.6) in the moment of preparation and after 3 days of settling at 298.15 K and 0.1 MPa.

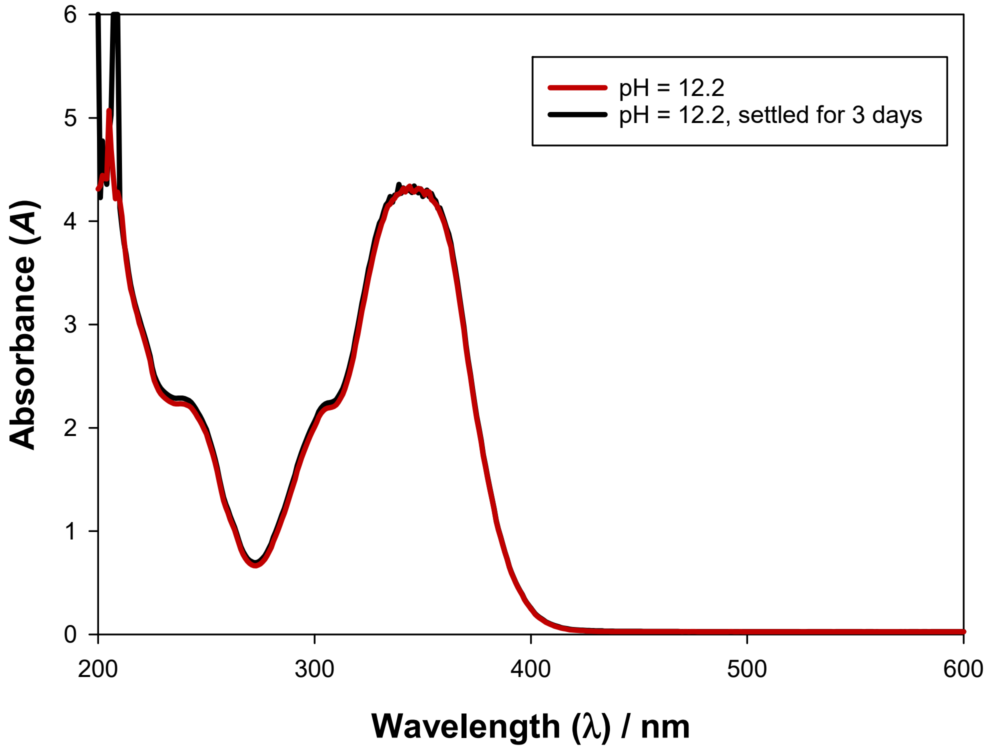

Figure S10. UV-Vis absorbance spectra of the aqueous stock solution of ferulic acid ($7.84 \cdot 10^{-5}$ $g \cdot mL^{-1}$ and pH = 12.2) in the moment of preparation and after 3 days of settling at 298.15 K and 0.1 MPa.

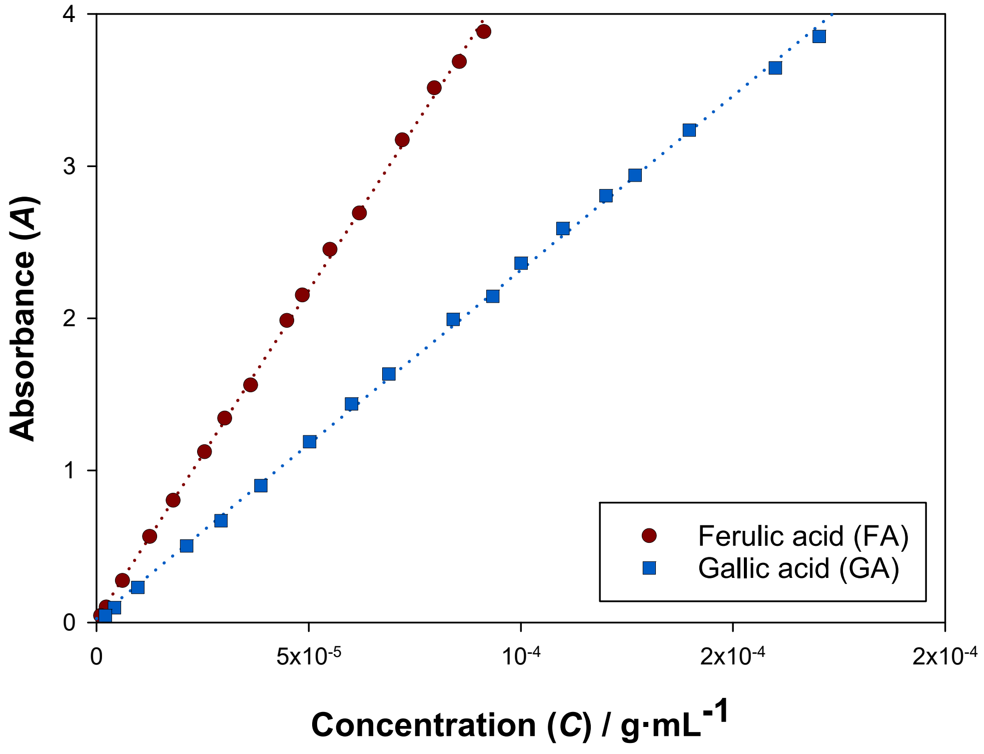

Figure S11. UV-Vis absorbance calibration curves for gallic acid ($\lambda = 260$ nm) and ferulic acid ($\lambda = 310$ nm) at pH = 7.5, T = 298.15 K and P = 0.1 MPa. The first-degree fittings follow equations: $A = 22\,917.3 \cdot C_{\mathrm{GA}}(\mathrm{g} \cdot \mathrm{mL}^{-1}) + 0.0252$ with a determination coefficient (R^2) of 0.9992 and $A = 43\,253.3 \cdot C_{\mathrm{FA}}(\mathrm{g} \cdot \mathrm{mL}^{-1}) + 0.0211$ with $R^2 = 0.9993$, respectively.

Table S1: Calculated mole fractions of each antioxidant stage and mean electrical charge (q) at different pH values for gallic acid (GA).

pH	x_{GA^0} [a]	$x_{GA^{-1}}$	$x_{GA^{-2}}$	$x_{GA^{-3}}$	q / e
0.00	1.00	0.00	0.00	0.00	0.00
0.25	1.00	0.00	0.00	0.00	0.00
0.50	1.00	0.00	0.00	0.00	0.00
0.75	1.00	0.00	0.00	0.00	0.00
1.00	1.00	0.00	0.00	0.00	0.00
1.25	1.00	0.00	0.00	0.00	0.00
1.50	1.00	0.00	0.00	0.00	0.00
1.75	1.00	0.00	0.00	0.00	0.00
2.00	0.99	0.01	0.00	0.00	-0.01
2.25	0.99	0.01	0.00	0.00	-0.01
2.50	0.98	0.02	0.00	0.00	-0.02
2.75	0.97	0.03	0.00	0.00	-0.03
3.00	0.95	0.05	0.00	0.00	-0.05
3.25	0.91	0.09	0.00	0.00	-0.09
3.50	0.86	0.14	0.00	0.00	-0.14
3.75	0.77	0.23	0.00	0.00	-0.23
4.00	0.66	0.34	0.00	0.00	-0.34
4.25	0.52	0.48	0.00	0.00	-0.48
4.50	0.38	0.62	0.00	0.00	-0.62
4.75	0.25	0.75	0.00	0.00	-0.75
5.00	0.16	0.84	0.00	0.00	-0.84
5.25	0.10	0.90	0.00	0.00	-0.90
5.50	0.06	0.94	0.00	0.00	-0.94
5.75	0.03	0.97	0.00	0.00	-0.97
6.00	0.02	0.98	0.00	0.00	-0.98
6.25	0.01	0.99	0.00	0.00	-0.99
6.50	0.01	0.99	0.01	0.00	-1.00
6.75	0.00	0.98	0.01	0.00	-1.01
7.00	0.00	0.97	0.02	0.00	-1.02
7.25	0.00	0.96	0.04	0.00	-1.04
7.50	0.00	0.93	0.07	0.00	-1.07
7.75	0.00	0.88	0.12	0.00	-1.12

8.00	0.00	0.81	0.19	0.00	-1.19
8.25	0.00	0.70	0.30	0.00	-1.30
8.50	0.00	0.57	0.43	0.00	-1.43
8.75	0.00	0.43	0.57	0.00	-1.57
9.00	0.00	0.29	0.71	0.00	-1.71
9.25	0.00	0.19	0.81	0.00	-1.81
9.50	0.00	0.12	0.88	0.00	-1.89
9.75	0.00	0.07	0.92	0.01	-1.94
10.00	0.00	0.04	0.95	0.01	-1.97
10.25	0.00	0.02	0.96	0.02	-2.00
10.50	0.00	0.01	0.95	0.04	-2.03
10.75	0.00	0.01	0.93	0.07	-2.06
11.00	0.00	0.00	0.88	0.11	-2.11
11.25	0.00	0.00	0.82	0.18	-2.18
11.50	0.00	0.00	0.71	0.28	-2.28
11.75	0.00	0.00	0.59	0.41	-2.41
12.00	0.00	0.00	0.44	0.56	-2.56
12.25	0.00	0.00	0.31	0.69	-2.69
12.50	0.00	0.00	0.20	0.80	-2.80
12.75	0.00	0.00	0.12	0.88	-2.88
13.00	0.00	0.00	0.07	0.93	-2.93
13.25	0.00	0.00	0.04	0.96	-2.96
13.50	0.00	0.00	0.02	0.98	-2.98
13.75	0.00	0.00	0.01	0.99	-2.99
14.00	0.00	0.00	0.01	0.99	-2.99

[a] x_{GA^0}, $x_{GA^{-1}}$, $x_{GA^{-2}}$ and $x_{GA^{-3}}$ are the mole fractions of gallic acid in the antioxidant stages of electrical charges equal to 0, -1, -2 and -3 e, respectively. e stands for the elementary charge ($1.602 \cdot 10^{-19}$ C).

Table S2: Calculated mole fractions of each antioxidant stage and mean electrical charge (q) at different pH values for ferulic acid (FA).

pH	x_{FA^0} [a]	$x_{FA^{-1}}$	$x_{FA^{-2}}$	q / e
0	1.00	0.00	0.00	0.00
0.25	1.00	0.00	0.00	0.00
0.5	1.00	0.00	0.00	0.00
0.75	1.00	0.00	0.00	0.00
1	1.00	0.00	0.00	0.00
1.25	1.00	0.00	0.00	0.00
1.5	1.00	0.00	0.00	0.00
1.75	1.00	0.00	0.00	0.00
2	1.00	0.00	0.00	0.00
2.25	0.99	0.01	0.00	-0.01
2.5	0.99	0.01	0.00	-0.01
2.75	0.98	0.02	0.00	-0.02
3	0.97	0.03	0.00	-0.03
3.25	0.95	0.05	0.00	-0.05
3.5	0.91	0.09	0.00	-0.09
3.75	0.85	0.15	0.00	-0.15
4	0.76	0.24	0.00	-0.24
4.25	0.64	0.36	0.00	-0.36
4.5	0.50	0.50	0.00	-0.50
4.75	0.36	0.64	0.00	-0.64
5	0.24	0.76	0.00	-0.76
5.25	0.15	0.85	0.00	-0.85
5.5	0.09	0.91	0.00	-0.91
5.75	0.05	0.95	0.00	-0.95
6	0.03	0.97	0.00	-0.97
6.25	0.02	0.98	0.00	-0.98
6.5	0.01	0.99	0.00	-0.99
6.75	0.01	0.99	0.01	-1.00
7	0.00	0.99	0.01	-1.01

7.25	0.00	0.98	0.02	-1.02
7.5	0.00	0.96	0.04	-1.04
7.75	0.00	0.94	0.06	-1.06
8	0.00	0.89	0.11	-1.11
8.25	0.00	0.82	0.18	-1.18
8.5	0.00	0.72	0.28	-1.28
8.75	0.00	0.60	0.40	-1.40
9	0.00	0.45	0.55	-1.55
9.25	0.00	0.32	0.68	-1.68
9.5	0.00	0.21	0.79	-1.79
9.75	0.00	0.13	0.87	-1.87
10	0.00	0.08	0.92	-1.92
10.25	0.00	0.04	0.96	-1.96
10.5	0.00	0.03	0.97	-1.97
10.75	0.00	0.01	0.99	-1.99
11	0.00	0.01	0.99	-1.99
11.25	0.00	0.00	1.00	-2.00
11.5	0.00	0.00	1.00	-2.00
11.75	0.00	0.00	1.00	-2.00
12	0.00	0.00	1.00	-2.00
12.25	0.00	0.00	1.00	-2.00
12.5	0.00	0.00	1.00	-2.00
12.75	0.00	0.00	1.00	-2.00
13	0.00	0.00	1.00	-2.00
13.25	0.00	0.00	1.00	-2.00
13.5	0.00	0.00	1.00	-2.00
13.75	0.00	0.00	1.00	-2.00
14	0.00	0.00	1.00	-2.00

[a] $x_{\mathrm{FA}^{0}}$, $x_{\mathrm{FA}^{-1}}$ and $x_{\mathrm{FA}^{-2}}$ are the mole fractions of ferulic acid in the antioxidant stages of electrical charges equal to 0, -1 and -2 e, respectively. e stands for the elementary charge ($1.602 \cdot 10^{-19}$ C).
